# Supplementary material for: Penalty kick or not? Differences in the interpretation of handball incidents in professional association football
Source: PLoS One. 2026 Feb 25;21(2):e0341822. doi: 10.1371/journal.pone.0341822 (PMC12935212; doi:10.1371/journal.pone.0341822)
Supplement: Attachment — Submitted filename: Response to Reviewers.pdf [file pone.0341822.s006.pdf]

EBERHARD KARLS
UNIVERSITÄT
TÜBINGEN

University of Tübingen · Wilhelmstraße 124 · 72072 Tübingen

Faculty of Economics and Social Sciences

Institute of Sports Science
Sport Psychology & Research Methods
Tobias Bauch, M.Sc.
tobias.bauch@dfb.de

PLOS One

December 27, 2025

Revision of the research article: "Penalty kick or not? Differences in the interpretation of handball incidents in professional association football"

Dear Editors and Reviewers,

thank you for your feedback on the original submission. The comments were very helpful, and we think by addressing them we could further increase the article's quality and relevance for the scientific community. Please find our responses to each point raised during the review process below:

Journal requirements:

- Please ensure that your manuscript meets PLOS ONE's style requirements, including those for file naming.
  → All style requirements were checked. The files belonging to the supporting information section were reuploaded with adapted file names.

- Please find our updated competing interests statement. Could you please change the online submission form on our behalf:

"The authors have declared that no competing interests exist.
Some of the authors are employed by the German Football Association (DFB). However, this affiliation did not influence the research process or outcomes. The study was designed and conducted independently, and none of the potential results would have conferred specific advantages or disadvantages to the authors or the DFB. This affiliation does not alter adherence to PLOS ONE policies on sharing data and materials."

- Please include your full ethics statement in the 'Methods' section of your manuscript file. In your statement, please include the full name of the IRB or ethics committee who approved or waived your study, as well as whether or not you obtained informed written or verbal consent. If consent was waived for your study, please include this information in your statement as well.
  → This information was included in the Methods section.

Reviewer #1:

- Errors in Table 4:
  → Indeed, two columns of the table were accidentally switched during the formatting of the document. We apologise for the mistake and thank you very much for pointing it out. The columns were reordered and all data and references in the text to it were checked.

- Scientific substantiation of the Discussion section:
  → The Introduction and Discussion sections were extended to cover additional aspects relevant to the scientific community.

Reviewer #2:

- L65-78 (and broader introduction/discussion sections): […] drawing on research in officiating other sports […] concepts such as decision-making thresholds, game management, and/or accuracy vs adequacy debate. […]
  → These topics were added to the introduction and discussion in the revised manuscript.

- L190: In your reporting, could you consider splitting the AREF group by separating assistant referees and VARs? This would provide an interesting comparison as their roles are very different, and the VARs would be experienced in watching video to make decisions, whether the assistant referees would not be.
  → This would be an interesting comparison indeed. Unfortunately, the number of specialised VARs was too small (n=8) to analyse them as a separate group. Therefore, these video assistants were included in the AREF group.

- L193: You could consider using the recently published officiating expert statement in your reporting, specifically performance level:
  Webb, T., Hancock, D. J., Weston, M., Warner, S., Helsen, W. F., MacMahon, C., ... & Tingle, J. K. (2025). The future for sport officiating research: an expert statement. Managing Sport and Leisure, 1-10.
  → This was considered in the methods section and an additional table with demographic information was added in the supporting information section.

- L210-212: Was the score and time of game included in the video clip? Either way, I would recommend discussing this in the discussion/limitations as to how the presence or absence of this information may have influenced the decision-making of the referees.
  → Yes, those were visible in the video clips. This aspect is now addressed in the limitations section.

- L218-219: Who conducted these interpretations - was it a member of the research team or an officiating expert? Or was this data made available to the research team?

→ The interpretations were done by the UEFA Referees Committee and available to the research team. This was clarified in the revised manuscript.

- L220-222: Could you explain this on-field review more? How were the videos occluded?
  → This was clarified in the revised manuscript.

- L223-225: Should this be reported in the results?
  → We believe this information should remain (only) in the Methods section for better clarity by addressing concerns of a potential bias due to the visibility of OFRs directly.

- L226-227: Do you have the data on how many times participants replayed the videos?
  → No, this information could unfortunately not be recorded in the technical environment we used.

- L235-236: Was this rating for videos in Study 2 based on the referees' rating in Study 1?
  → We clarified in the manuscript that the difficulty rating of 4.1 ± 2.6 refers to the data collected in Study 2.

- L486: This is a very interesting point. I would recommend the authors consider the implications around availability of VAR at different levels. For example, higher levels of officials have the luxury of using VAR that lower level officials do not. What recommendations would you have in your results for decision-making development of different levels of referees?
  → We considered this point thoroughly. However, we do not believe that the availability of the VAR has a direct influence on the results of our specific research question regarding handball interpretation. We added this aspect and our thoughts to the discussion.

- L494: You could also consider the following study as a discussion point for how officials apply decisions:
  Russell, S., Renshaw, I., & Davids, K. (2019). How interacting constraints shape emergent decision-making of national-level football referees. Qualitative Research in Sport, Exercise and Health, 11(4), 573-588.
  → This study is referenced now in the expanded introduction section.

We hope that the uploaded revision of the manuscript now fully meets PLOS ONE’s publication criteria and are looking forward to your response.

Yours sincerely,

Tobias Bauch, M.Sc.
Institute of Sports Science, University of Tübingen
DFB Schiri GmbH, Frankfurt am Main
